# Intercellular Interactions in Peripheral Venous Blood in Practically Healthy Residents of High Latitudes

**DOI:** 10.1155/2021/7086108

**Published:** 2021-09-02

**Authors:** L. K. Dobrodeeva, A. V. Samodova, S. N. Balashova, K. O. Pashinskaya

**Affiliations:** N. Laverov Federal Center for Integrated Arctic Research of the Ural Branch of the Russian Academy of Sciences, Arkhangelsk, Russia

## Abstract

The paper presents the results of studying the immunological parameters of 369 people who were practically healthy at the time of the survey, 298 women and 71 men, of which 216 people are living in the European North of the Russian Federation (173 women and 43 men) and 153 are residents of the Arctic (125 women and 28 men). The study was carried out in the morning (08:00–10:00 am). The study included the determination of the aggregation of erythrocytes, platelets, neutrophilic granulocytes, lymphocytes, hemogram study, hematological analysis, enzyme immunoassay, and flow cytometry. Statistical processing of the obtained data was carried out using the Statistica 7.0 software package (StatSoft, USA). It was found that the activity of aggregation of cells of peripheral venous blood in Arctic residents is 1.5–1.7 times higher than that in people living in more favourable climatic conditions. The frequency of registration of aggregation of erythrocytes and platelets is actually 2 times higher than the aggregation of leukocytes. Aggregation of erythrocytes is associated with an increase in the concentrations of transferrin and receptors for this transport protein. The frequency of detection of platelet aggregation is accompanied by an increase in transferrin concentrations; in cases of aggregation of nonnuclear blood cells, the content of NO_2_ in the blood serum is increased. Aggregation of neutrophilic granulocytes and lymphocytes is associated with an increase in the content of free adhesion molecules. Aggregation of erythrocytes and platelets is in evidence when it is necessary to trigger reactions of changes in the hemodynamics of microcirculation to increase the efficiency of oxygen and trophic supply of tissues. The adhesion of leukocytes to the endothelium determines the secretion of biologically active substances that contribute to a change in microcirculation and an increase in the migration of leukocytes into tissues for the implementation of phagocytic and cytolytic functions.

## 1. Introduction

Virtually, any cells, mobile and immobile, can interact with each other. The interaction of cells can be manifested by sticking of cells (adhesion), gluing of cells (conglutination, agglutination, and aggregation), or the formation of rosettes. It is possible that these are associated phenomena and show different functional levels of cell interaction. Adhesion, as a rule, is reversible; the reversibility of aggregation is determined by various conditions, either by the number of cells in the aggregate or by the concentration and type of enzymes secreted by the aggregate or by the degree of activation of plasma systems and the attraction of cytotoxic cells. The process of reaggregation is when cells of different tissues separated by treatment with various substances (for example, proteolytic enzymes or trypsin) are attached to each other forming aggregates characteristic only for a given population of cells in a particular tissue.

The purpose of cell contact is to ensure the passage of substances through the membrane that are not able to pass through it on their own. This intracellular mediator is cyclic AMP (cAMP). Adenylate cyclase converted into cAMP by ATP is localized in the plasma membrane and has a regulatory subunit on the outer surface and a catalytic subunit on the inner surface of the cell membrane. The interaction of the regulatory subunit with the extracellular factor stimulates or suppresses the activity of the catalytic subunit, which, upon stimulation, converts ATP into intracellular cyclic AMP (cAMP). Cyclic AMP is found in all cells; it is an independent intracellular primary hormone for the cell before the emergence of differentiated organisms. Its participation in the cell's responses to action across the membrane is not limited to its intracellular presence; the extracellular content of cAMP probably provides the morphological and functional unity of tissues and organs.

In response to any change in the intercellular environment which carries a potential threat of circulatory disorders, a reaction of the function of the microcirculatory unit develops with a change in blood volume and blood flow velocity. Cell adhesion to the endothelium is a signal for an urgent hemodynamic response [1–3]. Changes in hemodynamics are caused by an increase in shear stresses acting on the vessel wall [4].

A decrease in the size of the circulating pool of blood cells initiates the urgent secretion of serotonin and histamine, with the possible activation of the plasma systems of kinins and complement, as well as prostaglandins, if necessary [5–8]. In this phase, the adhesiveness of leukocytes increases and even a noticeable decrease in their concentration in the blood can be recorded.

Affected by histamine, during the first seconds, there is an almost complete inhibition of the electrical activity of the smooth muscles of the venules and a decrease in the blood filling of the vessel by 70–85%; subsequent vasodilation provides an increase in blood volume and acceleration of its fluidity. In this phase, the activity of adhesion and the reality of aggregation of blood cells are already sharply reduced and the venous outflow increases. Simultaneously with the dilatation of the vessels, there is a divergence of endothelial cells with the formation of gaps by contraction of the subplasmolemmal layer and then after 20 seconds by reducing the surface membrane of the endotheliocyte. The lifespan of histamine gaps or channels in the endothelium does not exceed 10 minutes [9, 10].

Violations of the contact interaction of cells cause changes in tissue permeability, including for large molecular structures. At the same time, vascular tone increases, shear stress increases, and the activity of transcapillary metabolism decreases. Nitric oxide inhibits the adhesion and aggregation of erythrocytes, platelets, and leukocytes. The additional synthesis of this vasodilator is stimulated by an increase in shear stress, bradykinin, thrombin, and mild hypoxia [11].

Endothelin I has a powerful vasoconstrictor effect [12, 13]. The effects of endothelin on various vasoactive substances are ambiguous and are determined by the receptor apparatus of the cell. Receptors A and В_2_ of myocytes mediate the vasoconstrictor effect of the paracrine action of endothelin, activating phospholipase C, while receptor В_1_ of endothelial cells stimulates the synthesis and secretion of NO, prostacyclin, and natriuretic peptide, i.e., vasodilation. The endothelium of the veins produces significantly more endothelin than the endothelium of the arteries [14]. Regulation of vasoconstriction and vasodilation by endothelin reduces the activity of adhesion and aggregation and activation of prothrombogenic, proliferative activity.

The complement system triggers the further continuation of the reaction; vasodilatation and increased vascular wall permeability reproduce the product of C1, C4, and C2 activation of the complement system (C-kinin). The complement system also supplies the main factor of chemotaxis, C5, which is formed by neutrophil enzymes and is enhanced by the reaction of platelets.

Restoration of the tone and permeability of the vascular wall caused by serotonin, histamine, kinins, or other agents is provided by catecholamines [15–18].

The content of the extracellular pool of signalling molecules, receptors, adhesion molecules, and their ligands in residents of high latitudes is significantly higher than in people living in more favourable climatic conditions. The dependence of this phenomenon on northern climatic conditions is demonstrated by the highest levels of the extracellular pool of various adhesion molecules in residents of the Arctic regions [19–22].

In connection with the above, we studied intercellular interactions and the frequency of registration of aggregation of peripheral venous blood cells in people living in the European North of the Russian Federation and in the Arctic.

## 2. Materials and Methods

The paper presents the results of studying the immunological parameters of 369 people who were practically healthy at the time of the survey, 298 women and 71 men, of which 216 people are living in the European North of the Russian Federation (173 women and 43 men) and 153 are residents of the Arctic (125 women and 28 men). All research was conducted with the consent of the volunteers and in accordance with the requirements of the World Medical Association's Declaration of Helsinki on the Ethical Principles of Medical Research (2000). The study was carried out in the morning (08:00–10:00 am). Vacuum systems of Vacutainer (Becton Dickinson International, USA) were used to collect venous blood. On the XS-500i hematological analyzer (Sysmex, Japan) in the peripheral venous blood of the subjects, the following were studied: WBC (total leukocyte count); erythrocyte parameters: RBC (total erythrocyte count), HGB (hemoglobin concentration), HCT (hematocrit—the proportion of blood volume occupied by erythrocytes), MCV (average erythrocyte volume in the total sample volume), MCH (average hemoglobin volume in RBC), MCHC (average concentration of hemoglobin in erythrocytes), RDW-SD (calculated width of distribution of red blood cells by volume, standard deviation), and RDW-CV (calculated width of distribution of red blood cells by volume, coefficient of variation); and platelet parameters: PLT (total platelet count), PDW (calculated platelet distribution width), MPV (mean platelet volume), P-LCR (large platelet ratio), and PCT (plateletcrit). Using a Nikon Eclipse Ni-U microscope (Japan), a hemogram in blood smears stained by the Romanowsky-Giemsa method was studied in peripheral venous blood. The aggregation of diluted blood erythrocytes was determined by the method of Levtov et al. [23] and microscopic observation in a thin blood smear [24, 25] and the aggregation of platelets and leukocytes by method of light microscopy in a blood smear [26, 27].

Concentrations of free molecule of intercellular adhesion-1 (sCD54), free transferrin receptor (sCD71), transferrin, and proinflammatory cytokine TNF-*α* were studied in blood serum by the method of enzyme-linked immunosorbent assay on the Evolis automatic enzyme-linked immunosorbent analyzer (Bio-Rad, Germany) with appropriate reagents (Bender MedSystems, Germany), irisin (BioVendor, Czech Republic), adrenaline and noradrenalin (IBL Germany), endothelin-1, total NO, endogenous NO_2_, and nitrate (NO_3_) (RnDSystems, USA). The content of lymphocyte phenotypes (CD3+, CD4+, CD8+, CD10+, CD16+, CD23+, CD25+, CD71+, CD95+, and HLA DR II) was determined in an indirect immunoperoxidase reaction using monoclonal antibodies (MedBioSpektr, Sorbent, Moscow) and by the method flow cytometry using an Epics XL apparatus from Beckman Coulter (USA) with ImmunoTech and Beckman Coulter Company (France) reagents. The concentration of circulating immune complexes (CIC) was investigated by the standard precipitation method using 3.5, 4.0, and 7.5% PEG-6000 in serum. The reaction was evaluated on the Evolis automatic enzyme immunoassay analyzer from Bio-Rad (Germany).

The mathematical analysis of the research results was carried out using the Microsoft Excel 2010 and Statistica 7.0 software packages (StatSoft, USA). The distribution laws of the values of immunological parameters were checked using the Pearson statistical test. Testing the null hypothesis of the equality of all means in the study groups was carried out using one-way analysis of variance. In conditions of disobedience of the data to the law of normal distribution, comparison of two different groups in terms of quantitative characteristics was carried out using the nonparametric Mann–Whitney *U* test. For each of the listed indicators, the parameters of descriptive statistics were calculated (*M* is the arithmetic mean, *σ* is the standard deviation, *m* is the standard error of the mean, Md is the median, *R* is the range, and *W* is the coefficient of variation, the boundaries of the 95% confidence interval). 0.05 was considered the critical level of significance (*p*).

## 3. Results

**Figure 1:**
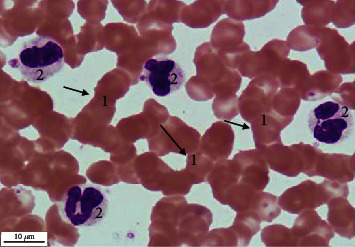
Aggregation of erythrocytes. Peripheral venous blood. Coloring according to Romanowsky-Giemsa. ×1000. 1: aggregation of erythrocytes; 2: neutrophilic granulocytes.

**Figure 2:**
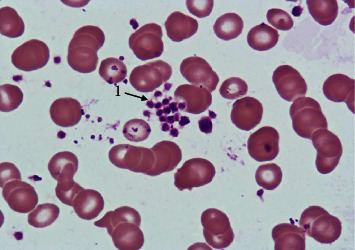
Platelet aggregation. Peripheral venous blood. Coloring according to Romanowsky-Giemsa. ×1000. 1: platelet aggregation.

**Figure 3:**
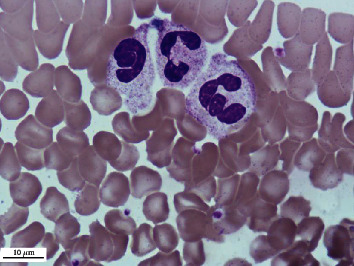
Aggregation of neutrophilic granulocytes. Peripheral venous blood. Coloring according to Romanowsky-Giemsa. ×1000.

**Figure 4:**
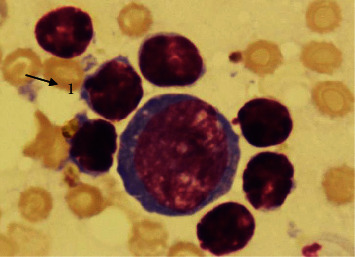
Aggregation of lymphocytes. Peripheral venous blood. Coloring according to Romanowsky-Giemsa. ×1000. 1: aggregation of lymphocytes.

**Figure 5:**
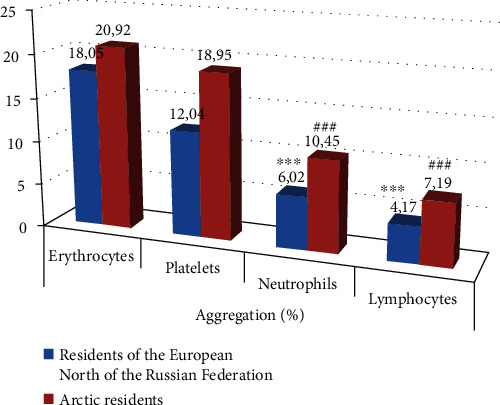
Frequency of registration of aggregation of peripheral venous blood cells in practically healthy residents living in the European North and the Arctic of the Russian Federation. ^∗∗∗,###^*p* < 0.001: reliability of differences when comparing indicators in the examined individuals with aggregation of erythrocytes and platelets.

A comparative study of the frequency of registration of aggregation of erythrocytes, platelets, neutrophilic granulocytes, and lymphocytes in practically healthy individuals living in different climatic conditions was of interest. It was found that the frequency of detection of aggregation of erythrocytes (Figure 1), platelets (Figure 2), neutrophilic granulocytes (Figure 3), and lymphocytes (Figure 4) in the venous blood of practically healthy individuals living in the Arctic is 1.5–1.7 times higher compared with the level of aggregation of blood cells in individuals living in the European North of the Russian Federation. In practically healthy individuals living in the Arctic, most often aggregate erythrocytes and platelets (20.92% and 18.95%). Peripheral blood leukocytes form aggregates almost 2 times less often (neutrophilic granulocytes in 10.45%, lymphocytes in 7.19%) (Figure 5).

It was found that during the polar night, the frequency of registration of erythrocyte aggregation in residents of the Arctic is higher (16.12 and 24.40%) and the hemoglobin level decreases in parallel (from 138.32 ± 4.56 to 115.28 ± 4.12 g/l, *p* < 0.01).

A comparative analysis of the frequency of detecting aggregation of blood cells and their content, as well as the concentrations of biologically active substances and phenotypes of lymphocytes in the inhabitants of the Arctic, revealed some relationships (Table 1).

Aggregations of erythrocytes and platelets coincide in 6 people out of 29 (20.7%), a combination of aggregation of neutrophilic granulocytes and erythrocytes (2 cases out of 15), neutrophilic granulocytes and platelets (1 out of 15), and lymphocytes and nonnuclear blood cells (2 of 11) were rare.

Aggregation of erythrocytes is associated with an increase in the content of the phenotype with the transferrin receptor (CD71+) and its free receptor (sCD71) in blood serum, transferrin, endogenous NO_2_, and TNF-*α*. With platelet aggregation, the content of transferrin and endogenous NO_2_ is higher (Table 1).

Platelet aggregation is associated with higher blood concentrations of transferrin and endogenous NO_2_ (Table 1).

Among all the parameters studied by us, the level of neutrophil aggregation activity was associated with a decrease in the content of these cells in the circulating blood and an increase in the concentration of adhesion molecules sCD54 (Table 1).

Aggregation of lymphocytes in circulating blood is the rarest in comparison with that of erythrocytes, platelets, and neutrophils. The presence of aggregation of lymphocytes, as well as aggregation of neutrophils, is associated with an increase in the serum content of the adhesion molecule sCD54 (Table 1).

## 4. Discussion

Transferrin is a *β*-globulin, at both ends of the molecule of which there are glycoproteins that can hold 2 atoms of 3-valent iron. The carbohydrate part of transferrin contains 1.4-2% of sialic acid, and depending on its content, the types of ligand are differentiated [28]. Usually, transferrin iron saturation is incomplete; unsaturated or latent iron-binding capacity indicates a reserve capacity to bind Fe. Fe^3+^ ions during their transfer to the small intestine and neutralization undergo polymerization and precipitate in the form of Fe(OH)_3_ which is absorbed; Fe^2+^ ions do not polymerize and therefore are better absorbed.

The main function of transferrin is to transport Fe^3+^ from the intestinal mucosa and spleen sinuses to the bone marrow where it is utilized in the process of hemoglobin synthesis. The increased transferrin erythroid cell affinity has been experimentally proven using labelled transferrin. Intracellular Fe is found in mitochondria where the metal is used to synthesize various enzymes; in the mitochondria of erythrocytes, Fe binds to protoporphyrin and turns into heme. Fe is deposited in the cell in the form of ferritin—a water-soluble complex of Fe hydroxide with the protein apoferritin. Hemosiderin is characterized by the absence of a part of the protein and is insoluble, but both of these proteins are immunologically identical. Metal reserves are used very slowly; a decrease in the activity of using the Fe reserve is noted in infections and inflammatory processes, especially chronic, malignant neoplasms.

As a result, under these conditions, the reserves of deposited Fe in the reticuloendothelial system (RES) increase. Noticeable reductions in the supply of metal are rarely observed during starvation and in the absence of iron intake from food. The disappearance of hemosiderin granularity in RES cells and erythroid elements is a clear sign of iron deficiency in the body. In case of blood loss, pregnancy, and breastfeeding, the balance of iron content in the body is regulated primarily by an increase in intestinal absorption and an acceleration of plasma Fe turnover due to disintegrating erythrocytes in their stable depots [29].

Individuals living in northern climatic conditions unfavourable for humans have a lower life expectancy of erythrocytes and lower average hemoglobin content in red blood cells with increased fetal hemoglobin concentration [30–33].

Changes in the shape of a carrier cell of 0_2_ and its lipid metabolism and an increase in the viscosity of cell membranes are factors that affect circulation, the rate of diffusion of 0_2_, and gas exchange [30, 31, 34–36].

An increase in the microviscosity of lipids in the erythrocyte membrane with an increase in cholesterol and monounsaturated fatty acids slows down the release of О_2_ from the erythrocyte, impairs the rheological properties of blood, and reduces the rate of intracellular Hb deoxygenation [30, 31, 34–38]. The development of northern tissue hypoxia is characterized by changes at all stages of 0_2_ delivery, from external respiration to its consumption by tissues [39].

Since capillaries are devoid of contractile elements, the efficiency of oxygen and trophic supply of tissues mainly depends on the rheological properties of blood and blood viscosity becomes a determining factor in ensuring tissue perfusion. Erythrocytes, accounting for almost half of the blood volume (40–45%), determine the fluidity of the blood and play a major role in the formation of its rheological properties. The viscosity of blood is due to the forces of inertia and cohesion and is determined mainly by the concentration of blood corpuscles (hematocrit), aggregation, and deformation of erythrocytes. Aggregation of erythrocytes is the main determinant of blood viscosity in physiological conditions and in pathology causing changes in microcirculation [40, 41].

The formation of erythrocyte aggregates changes the blood flow velocity and the distribution of linear velocities along the diameter of the vessel [1–3]. Changes in hemodynamics are caused by an increase in shear stresses acting on the vessel wall [4]. “Coin bars” appear with an increase in the content of serotonin, histamine, prostaglandins, kinin, and acetylcholine and are associated with a slowdown in the blood flow rate in the capillaries [42, 43].

Thus, the aggregation of erythrocytes associated with an increase in the content of transferrin and transferrin receptors most likely reflects a complex response to an increase in the activity of utilization of iron released during the destruction of erythrocytes in the spleen and the formation of an adoptive hemodynamic microcirculation regime to ensure the optimal efficiency of oxygen and trophic supply of fabrics.

The transferrin receptor is present on a wide variety of cells; it appears most actively when activated and dividing cells need additional iron production [44–50]. Transferrin is known to stimulate IL-2 production, DNA synthesis, and cell proliferation [51].

An increase in the content of cells with a membrane receptor for transferrin and, in parallel, its free form in plasma reflects the activation of cells [52–57].

Under conditions of increased demand or deficiency of iron, cells even begin autonomous synthesis of transferrin which is regulated by the proinflammatory cytokines IL-1, IL-2, IL-6, and TNF-*α*, as well as NO. Proinflammatory cytokines increase the production of NO in the cell which enhances tissue blood flow, plasma exudation, and cell proliferation. The involvement of TNF-*α* in these reactions may be associated with the synthesis of prostaglandin E2 and F1, as well as leukotriene B4 [58, 59].

It is known that red blood cell clumps are often found within the flowing blood. The ability of erythrocytes to move in a cluster was discovered by William Hewson in as early as 1773 [60]. Aggregation of erythrocytes is an initially normal process and a consequence of the action of blood flow forces, which is substantiated by the classical works of Fahraus [61] and Schmid-Schönbein et al. [62]. Disaggregation of normal erythrocytes occurs relatively easily as a result of mutual sliding of cell surfaces in opposite directions [63]; in arterial blood, erythrocyte aggregates are separated near the vascular wall [64].

An increase in the severity and duration of aggregation creates a risk of pathological development. The consequences of pathological aggregation are intravascular disorders of microcirculation (slowing down to stasis, turbulent blood flow and inclusion of arteriovenular shunts), hypoxia and acidosis in tissues and organs, and metabolic disorders in tissues with the development of various dystrophies [65].

Platelets provide an adhesive-aggregation function by forming a primary platelet plug in a bleeding vessel; deliver plasma coagulation factors to the bleeding site; perform a coagulation function of blood coagulation, retraction, and coagulation of a clot; and also participate in maintaining the structural integrity and normal vascular permeability performing angiotrophic function.

The mechanism of platelet hemostasis, generalized for the first time in the scheme of the International Committee on Hemostasis and Thrombosis, determines the primary adhesion of platelets and their spreading by the first stage of platelet plug formation. This is followed by the release from the adhered platelets of ADP, serotonin, histamine, and platelet coagulation factors. Next comes the attraction of other platelets to aggregation under the influence of the products secreted by them, which is initially reversible; with an increase in the size of the aggregate, viscous metamorphosis develops under the influence of the thrombin formed in this aggregate and the aggregation becomes already irreversible. The retraction of the clot occurs due to the activation of the thrombostenin-ATP system in platelets [66].

They initiate platelet adhesion (serotonin), adhesion and reversible aggregation (serotonin and ATP), and adhesion and the reaction of releasing the contents of granules, thromboxane, and prostaglandins (ADP). Serotonin, a weak inducer, induces cell reshaping, spreading, and reversible aggregation. Thrombin and collagen determine all stages of platelet secretory activity with secretion from all types of dense granules [67].

The nitric oxide cycle forms the absorption phase, and in the absence of nitric oxide secretion and absorption, endothelial cells constantly secrete endothelin vasoconstrictors. The lack of vasodilation occurs as a result of the predominance of vasoconstrictors, mainly endothelin-1.

Changes in the regulation of blood flow in the microvasculature can lead to a redistribution of blood flows in microvessels with a dominance of the shunt component and a decrease in nutritional perfusion. A smaller increase in nutritional perfusion, typical for northerners, indicates a possible risk of the deficiency of endothelium-dependent vasodilation as a result of a shift in the balance of nitric oxide synthesis and vasoconstrictors (endothelin-1) towards the latter's dominance.

An increase in the activity of platelet aggregation in northerners during the polar night, which is more significant in residents of the Arctic, can inhibit the adhesiveness of the endothelium. It is known that platelets lie at the base of the parietal thrombi but this surface becomes nonadhesive for new portions of circulating platelets [68]. Thus, the spreading of platelets along the endothelium can create a nonadhesive shield for endothelial cells. In general, the reversible aggregation of platelets, as well as of erythrocytes, ensures the formation of optimal microcirculation conditions for effective oxygen and trophic supply of tissues.

Aggregation of neutrophilic granulocytes and lymphocytes is associated with an increase in the content of free adhesion molecules. The adhesion of leukocytes to the endothelium determines the secretion of biologically active substances that contribute to a change in microcirculation and an increase in the migration of leukocytes into tissues for the implementation of phagocytic and cytolytic functions. The increased need for utilization of metabolic products in tissues among residents of the northern territories is due to a more significant metabolic cost.

Neutrophils normally have a higher viscosity compared to the level of this property in erythrocytes and platelets. Direct interaction of the agent with the plasmolemma or indirect interaction of the ligand and the neutrophil receptor occurs in less than 30 seconds and is accompanied by an increase in membrane fluidity due to its enrichment with unsaturated fatty acids [69, 70].

Immediately after the interaction of the neutrophilic granulocyte with the membrane, Са^2+^ is lost and its entry increases [71–73]; an increase in the accumulation of Са^2+^ occurs simultaneously with the release of lysosomes and the loss of the ATP content.

Attachment of leukocytes by pseudopodia to the surface of the endothelium or to the extracellular matrix makes it possible to move by pulling the cell towards the pseudopod; simultaneously, specific neutrophilic granules of the leukocyte move to the area of pseudopodia. Neutrophilic neutrophil granules include lysozyme, lactoferrin, and alkaline phosphatase which are active only in a neutral environment.

Tissue hypoxia with a decrease in the partial pressure of О_2_ ang changes in microcirculation and vascular permeability cause an increased level of neutrophil adhesion in venules [74–76]. The adhesiveness of blood cells of leukocytes is enhanced by a whole series of biologically active substances produced by the activated neutrophils themselves [77].

Adhesion and release of specific leukocyte granules into the pseudopodia region are associated with an increase in the secretion of adhesion molecules, selectins, their ligands, and chemotactic receptors in the leukocyte adhesion region [78–82].

A higher level of adhesion activity and subsequent migration of activated neutrophilic granulocytes into the tissues of the inhabitants of the north is necessary due to the low level of clearance of metabolic products which is proved by the predominance of the intake of protein and fluid from the blood into the tissue over the activity of their excretion in northerners [39]. Neutrophils are involved in the clearance of waste products of cells and their apoptosis. Phagocytosis of apoptotic bodies by granulocytes occurs very quickly and does not cause signs of inflammation [83, 84]. A high level of chemoattractants promotes a constant migratory flow of granulocytes, which is often manifested by a decrease in the content of circulating and actively phagocytic neutrophils [85]. In the presence of a sufficient concentration gradient of the chemoattractant, the location of receptors on the cell membrane surface becomes asymmetric, concentrates at one of the poles in the form of a cap (capping), and determines the direction of its movement [86].

Irreversible aggregation of neutrophils is involved in the formation of leukostasis and leukopenia due to a decrease in the content of cells in the circulating pool and their transition to a marginal one. A high level of aggregation (clumping) with the participation of C5a is already associated with lysis of neutrophils and can lead to granulocytopenia. Lysis of the neutrophil aggregate was reproduced under experimental conditions in capillaries in vitro [5].

Lymphocytes are unique cells that recirculate from the tissue space to the lymphoid organs, with the exception of the thymus and bone marrow, where lymphocytes do not reenter [87]. The movement of lymphocytes from tissues back into the blood is considered a rapid type of migration and takes only a few hours (depending on the distance, 0.6–5 hours).

Lymphocytes adhere to the endothelial lining of microvessels and penetrate through the endothelium, as a rule, following a decrease in the activity of the transition of polynuclear leukocytes. Apparently, the penetration of lymphocytes is somehow prepared by the migration of polynuclear cells. It is known that a weakly alkaline environment promotes the activity of lymphocytes, while an acidic environment inhibits it. It is likely that the earlier activation of neutrophils with the release of enzymes of neutrophil granules, which alkalize the medium, contributes to the activation of lymphocytes for adhesion and migration. Neutrophils are capable of intraphagosomal and extracellular degranulation; therefore, they can regulate the functional activity of many cells, including lymphocytes, at the autocrine and paracrine levels [88–94].

Indeed, in the lymphatic formations closest to the area of irritation or damage, vascular congestion and infiltration of the vascular wall by segmented granulocytes are observed [95–97]. Already after 24 hours, the maximum number of neutrophilic granulocytes is recorded in the regional vessels, which, leaving the dilated blood vessels, fill the marginal and intermediate sinuses, trabeculae, and pulp cords, accounting for up to 30% of the cellular composition [95, 96].

Considerable interest in the phenomenon of lymphocyte adhesion is due to the interrelationships between the activity of cell adhesion, their blast transformation, and mitosis. If, during the aggregation of lymphocytes, one molecule of the receptor structure binds to two cells, then to initiate mitosis, two molecules located on the same membrane need to crosslink with each other. The precipitated lymphocyte receptors, like in other cell types, can be shed or pinocytosed [98].

The transfer of lymphocytes from the lymph to the bloodstream of the lymph nodes and back from it to the blood occurs at the level of postcapillary venules, which is associated with the special properties of their endothelial lining, in particular with the presence of wide interendothelial fissures [99–102].

## 5. Conclusion

So, the activity of aggregation of peripheral venous blood cells in residents of the Arctic is 1.5–1.7 times higher than that in people living in more favourable climatic conditions. The frequency of registration of aggregation of erythrocytes and platelets is actually 2 times higher than the aggregation of leukocytes. Aggregation of erythrocytes is associated with an increase in the concentrations of transferrin and receptors for this transport protein. The frequency of detection of platelet aggregation is accompanied by an increase in transferrin concentrations; in cases of aggregation of nonnuclear blood cells, the content of NO_2_ in blood serum is increased. Thus, the aggregation of erythrocytes and platelets is manifested when it is necessary to trigger reactions of changes in the hemodynamics of microcirculation to increase the efficiency of oxygen and trophic supply of tissues.

Adhesion is the initial stage of a general biological reaction which transforms a set of elements, organs, and tissues into a system by means of intercellular contacts. A wide variety of factors that change the surface tension of cells, transmembrane potential, and membrane receptor structures cause adhesion and subsequent aggregation of cells. The binding of receptors to each other increases the activity of the membrane with an increase in the adhesive properties of the cell, its mobility, intercellular interaction, and its functional activity.

The role of specific cell adhesion in the spatial organization of tissues is confirmed by the ability of some tissue cells like histotypic aggregation and reaggregation [103–105].

## Figures and Tables

**Table 1:** Relationship between the frequency of aggregation of peripheral venous blood cells and immune parameters in practically healthy residents of high latitudes (*M* ± *m*).

Studied parameters	Registration of the presence of an aggregation of the examined persons (*n* = 153)			
	Erythrocytes	Platelets	Neutrophils	Lymphocytes
Aggregation detection rate (%)	20.92 (32)	18.95 (29)	10.45 (15)	7.19 (11)
Erythrocytes (10^12^ cells/l)	4.28 ± 0.27	4.32 ± 0.30	4.63 ± 0.25^∗∗^	4.51 ± 0.28^∗^
Platelets (10^9^ cells/l)	275.32 ± 30.24	289.53 ± 27.62	262.83 ± 24.02^∗∗^	258.36 ± 26.54^∗∗^
Monocytes (10^9^ cells/l)	0.23 ± 0.08	0.21 ± 0.09	0.16 ± 0.08	0.18 ± 0.09
Neutrophilic granulocytes (10^9^ cells/l)	2.48 ± 0.15	2.36 ± 0.18	2.07 ± 0.24^∗∗^	2.37 ± 0.19
Lymphocytes (10^9^ cells/l)	2.19 ± 0.27	2.16 ± 0.21	2.17 ± 0.25	2.18 ± 0.24
CD3+ (10^9^ cells/l)	0.61 ± 0.09	0.62 ± 0.10	0.65 ± 0.12	0.61 ± 0.17
CD10+ (10^9^ cells/l)	0.44 ± 0.10	0.42 ± 0.09	0.43 ± 0.11	0.45 ± 0.08
CD23+ (10^9^ cells/l)	0.40 ± 0.08	0.39 ± 0.09	0.32 ± 0.10	0.30 ± 0.08
CD71+ (10^9^ cells/l)	0.50 ± 0.09	0.32 ± 0.07^∗^	0.33 ± 0.10^∗^	0.34 ± 0.12^∗^
sCD71+ (ng/ml)	6187 ± 224.6	4991 ± 248.1^∗∗^	5452 ± 252.2^∗∗^	5136 ± 268.4^∗∗^
Transferrin (mg/ml)	1487.8 ± 262.6	1657.1 ± 334.5	1169.1 ± 337.6^∗∗^	1169.1 ± 337.6^∗∗^
Adrenaline (pg/ml)	30.67 ± 2.11	30.31 ± 2.04	30.82 ± 2.68	30.56 ± 2.16
Endothelin-1 (fmol/ml)	1.91 ± 0.42	1.87 ± 0.48	1.89 ± 0.39	1.95 ± 0.54
Irisin (*μ*g/ml)	2.28 ± 0.87	2.75 ± 0.69	2.77 ± 0.74	2.56 ± 0.92
Total NO (*μ*mol/l)	27.75 ± 1.23	27.17 ± 1.44	27.57 ± 1.69	26.89 ± 1.71
Endogenous NO_2_ (*μ*mol/l)	14.51 ± 3.25	14.62 ± 2.42	10.74 ± 2.46^∗∗^	10.21 ± 2.30^∗∗^
Нитрат (NO_3_) (*μ*mol/l)	15.03 ± 3.14	14.97 ± 3.63	16.28 ± 3.48^∗∗^	16.85 ± 3.58^∗∗^
sCD54 (ng/ml)	202.51 ± 28.79	203.43 ± 24.81	268.73 ± 27.18^∗∗^	245.34 ± 28.45^∗∗^
TNF-*α* (pg/ml)	14.05 ± 1.49	7.08 ± 1.65^∗∗∗^	6.63 ± 1.55^∗∗∗^	8.21 ± 1.73^∗∗∗^
CD95+ (10^9^ cells/l)	0.34 ± 0.8	0.33 ± 0.09	0.37 ± 0.10	0.35 ± 0.11
CIC IgG (g/l)	45.32 ± 4.62	46.78 ± 4.86	42.65 ± 4.24	45.43 ± 4.46

^∗^*p* < 0.05; ^∗∗^*p* < 0.01; ^∗∗∗^*p* < 0.001: reliability of differences when comparing indicators in the examined individuals with erythrocyte aggregation.

## Data Availability

The data for the study were patented at the Federal Institute of Industrial Property: (1) immunological blood parameters of residents of the village of Revda, Murmansk region/Dobrodeeva L. K., Patrakeeva V. P., Samodova A.V., and Shashkova E. Yu./certificate of registration of the database no. 2018621014, 06.07.2018; (2) immunological parameters of venous and capillary blood before and after a single cold exposure during the minimum duration of daylight in residents of Arkhangelsk/Dobrodeeva L. K., Samodova A.V., Patrakeeva V. P., Stavinskaya O. A., Balashova S. N., Pashinskaya K. O., and Basova E. E./certificate of registration of the database no. 2018621015, 06.07.2018; (3) immunological parameters of venous and capillary blood of residents of the village of Barentsburg arch. Svalbard/Samodova A.V., Shtaborov V. A., Pashinskaya K. O., and Dobrodeeva L. K./certificate of registration of the database RU 2020620494, 17.03.2020, application no. 2020620310 of 04.03.2020; and (4) indicators of cellular and local immunity in residents living in the village of Lovozero of the Murmansk region/Shtaborov V. A., Shashkova E. Yu., Pashinskaya K. O., Basova E. E., Dobrodeeva L. K., and Samodova A.V./certificate of registration of the database no. 2020620495, 17.03.2020, application no. 2020620309 dated 04.03.2020.
